# Dietary Factors and Dental Caries among Adolescents: Eight-Year Follow-up Study

**DOI:** 10.1177/23800844251314856

**Published:** 2025-02-24

**Authors:** M. Methuen, V.F. Kukkonen, V. Anttonen, S. Mikkonen, J. Väistö, S. Soininen, M. Närhi, T.A. Lakka, A.L. Suominen, A.-M. Eloranta

**Affiliations:** 1 Institute of Dentistry, Faculty of Health Sciences, University of Eastern Finland, Kuopio, Finland; 2 Oral and Maxillofacial Diseases Teaching Unit, Kuopio University Hospital, Finland; 3 Department of Oral and Maxillofacial Diseases, Kuopio University Hospital, Finland; 4 Research Unit of Population Based Studies, University of Oulu, Finland; 5 MRC, Oulu University Hospital and University of Oulu, Finland; 6 Department of Environmental and Biological Sciences, University of Eastern Finland, Kuopio, Finland; 7 Department of Technical Physics, University of Eastern Finland, Kuopio, Finland; 8 Institute of Biomedicine, School of Medicine, University of Eastern Finland, Kuopio, Finland; 9 Physician and Nursing Services, Health and Social Services Centre, Wellbeing Services County of North Savo, Varkaus, Finland; 10 Department of Clinical Physiology and Nuclear Medicine, Kuopio University Hospital, Kuopio, Finland; 11 Foundation for Research in Health Exercise and Nutrition, Kuopio Research Institute of Exercise Medicine, Kuopio, Finland

**Keywords:** dental decay, caries experience, diet, nutrition, dietary behavior, child, teenagers

## Abstract

**Objective:**

The aim of this study was to conduct a longitudinal investigation of the associations between changes in dietary factors and changes in caries experience among Finnish children and adolescents participating in the Physical Activity and Nutrition in Children (PANIC) study.

**Methods:**

Among 487 children included at baseline at the age of 6 to 8 y, 406 were reexamined at 2-y follow-up and 202 at 8-y follow-up. Food consumption, nutrient intake, and eating frequency were assessed using 4-d food records; diet quality was assessed using the Baltic Sea Diet Score; and eating behavior was evaluated using the Children’s and Adult’s Eating Behaviour Questionnaires. At baseline and 2-y follow-up, caries findings were recorded using the World Health Organization guidelines and at 8-y follow-up using the International Caries Detection and Assessment System criteria. Generalized linear mixed-effects regression analyses were used.

**Results:**

Over 8 y from childhood to adolescence, improved diet quality (β = −0.017, P = 0.046) and increased consumption of butter and butter-oil mixtures (β = −0.009, P = 0.044) were associated with decreased caries experience. Increased number of snacks (β = 0.072, P = 0.032), increased consumption of sour milk products (β = 0.001, P = 0.039) and salty snacks (β = 0.006, P = 0.010), and increased calcium intake (β = 2.41 × 10^−4^, P = 0.022) were associated with increased caries experience. However, the latter association was explained by the consumption of sour milk products (β = 1.88 × 10^−4^, P = 0.090). Increased enjoyment of food was associated with decreased caries experience (β = −0.121, P = 0.046), and increased slowness in eating (β = 0.113, P = 0.051) and food fussiness (β = 0.140, P = 0.009) were associated with increased caries experience.

**Conclusions:**

A healthy diet is vital for oral health among children and adolescents. Dietary behaviors developing from childhood to adolescence seem to be associated with caries experience in adolescence. Dietary counseling aimed at improving dental health from childhood to adolescence should include avoiding frequent snacking, strengthening healthy eating behavior, and composing good overall diet quality.

**Knowledge Transfer Statement:**

Results of this longitudinal study showed how crucial a healthy diet is for oral health among growing children. Eating behaviors and enjoyment of food play also a role in maintaining good oral health. Research results can be used when planning dietary recommendations and health education for children and adolescents.

## Introduction

Diet plays a major role in the etiology of dental caries, with fermentable sugars as the main clearly documented causative factor (Gustafsson et al. 1954). The principal risk factor for the manifestation of caries disease is high and frequent sugar consumption (Gustafsson et al. 1954; Bernabé et al. 2016), which, under ecological imbalances, creates a state of dysbiosis in the dental biofilm (Giacaman 2018). Consequently, diet-derived sugars undergo anaerobic metabolism, forming dental plaque, wherein bacteria-formed organic acids demineralize dental hard tissues, causing caries lesions to develop.

The role of diet in the caries process may not be restricted to only fermentable sugars. By following a diet containing only a limited amount of fermentable carbohydrates, an ecological change toward an anticariotic microbiome is also possible. Fruits, berries, vegetables, high-fiber grain products, and milk may even have caries-protective effects (Moynihan 2007; Giacaman 2018). These food items and fish are key ingredients in the Baltic Sea diet (BSD), on which Scandinavian countries have based their dietary recommendations (Kanerva et al. 2014). However, studies on the association between BSD and caries are sparse. Several factors influence the processing of carbohydrates in an oral cavity. Certain fatty acids, proteins, and polyphenols seem to counteract the effect of sugars on caries through several antibacterial mechanisms (Desbois and Smith 2010; Giacaman 2018). Again, diet cariogenicity increases when fermentable sugars are consumed together with complex and processed carbohydrates such as starch (Giacaman 2018). Moreover, eating behaviors can affect oral health. For example, emotional eating has been associated with the presence of negative emotions such as anxiety and depression (Goossens et al. 2009) and can lead to eating disorders (Whiteside et al. 2007). Anxiety, depression, and eating disorders can significantly deteriorate oral health (Kisely et al. 2016).

Only few longitudinal studies have monitored changes in diet quality and eating behaviors associated with changes in caries status among young people (Virkkala et al. 2022; van Meijeren-van Lunteren et al. 2023). Previously we reported the association of better diet quality and also some health-promoting eating behaviors with decreased caries experience among children, when other eating behaviors, such as food fussiness, increased caries experience (Virkkala et al. 2022). The aim here is to investigate the longitudinal associations of changes in eating frequency, energy and nutrient intake, diet quality, food consumption, and eating behaviors with a change in caries experience from childhood to adolescence.

## Methods

### Study Design

The Physical Activity and Nutrition in Children (PANIC) study is an intervention study with an ongoing follow-up in a child population sample from the Kuopio city, Finland. Altogether, 736 children aged 6 to 8 y starting the first grade in primary schools between 2007 and 2009 were invited to participate in the study. The participants were divided into an intervention and a control group. The intervention included 6 physical activity and dietary counseling sessions during the 2-y follow-up (0.5, 1.5, 3, 6, 12, and 18 mo after baseline). Each intervention visit included 30 to 45 min of physical activity counseling and 30 to 45 min of dietary counseling for the children and their parents or caregivers, as described in detail before (Lakka et al. 2020). The control group received general verbal and written advice on health-improving physical activity and diet at baseline but no active intervention. After the 2-y follow-up examinations, the diet and physical activity intervention was continued less intensively with 1 counseling session per year until the 8-y follow-up examinations (24, 36, 48, 60, 72, 84, and 96 mo after the baseline). The session consisted of 30 min of diet counseling and 30 min of physical activity counseling. Data for the present analyses were collected between 2007 and 2018 and were received from baseline (*n* = 487), 2-y follow-up (*n* = 406), and 8-y follow-up (*n* = 202) examinations. The research protocol has been described in detail earlier (Lakka et al. 2020; Virkkala et al. 2022).

### Assessment of Dental Caries

#### Baseline and 2-y follow-up

At baseline and 2-y follow-up, dental examinations were done as suggested by the World Health Organization (1997) in primary dental care clinics. Experienced dentists recorded teeth as having initial lesions (I), being decayed (D), missing (M), or/and having fillings (F). Caries lesions were examined visual-tactilely from 5 tooth surfaces, aided by fiber-optic transillumination (FOTI), which is commonly used in Finnish clinical practice. Initial lesions were defined as lesions limited to the enamel and decayed as lesions extended to the dentin. Indices for decayed, missing, and filled permanent/primary teeth (DMFT/dmft) and tooth surfaces (DMFS/dmfs) were calculated. Missing teeth due to exfoliation were not separated from the primary tooth indices. Data were collected from electric patient files of Kuopio city with the permission of the record keeper.

#### 8-y follow-up

At 8-y follow-up, dental examinations were carried out in the dental clinic of the University of Eastern Finland by an experienced dentist (M.M.). Before the field phase of the 8-y follow-up study, the researcher (M.M.) was trained and calibrated for examinations by V.A., who was familiar with similar studies (Tanner et al. 2013; Karki et al. 2018). Images of teeth were used to demonstrate caries lesions according to the International Caries Detection and Assessment System (ICDAS) (Ismail et al. 2007; ICDAS Coordinating Committee 2012) (Fig.). During the 2-d training, 6 patients were examined together with M.M. and V.A. to ensure the acceptability of the protocol and criteria. Because the field phase took such a long time (24 mo), training and calibration were repeated every 3 mo to maintain the standards. On those occasions, any problems concerning the data collection were discussed. To investigate the reliability of the examinations, parallel examinations were carried out among 13 adolescents during those retraining sessions. The interexaminer agreement was moderate with a Cohen’s kappa value of 0.574 (minimum 0.293, maximum 0.811) for dentinal lesions, whereas for sound surfaces, the kappa value was 1.0.

**Figure.**
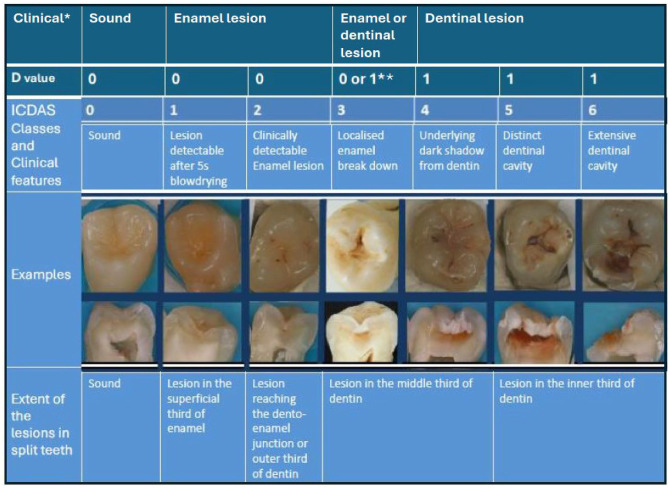
International Caries Detection and Assessment System (ICDAS) classification of carious lesions and corresponding D-values from DMF (decayed, missing, filled) classification. *Clinical classification used in Finland. **ICDAS 3 lesions are sometimes recognized as sound, sometimes as decayed. In this study, active ICDAS 3 lesions are considered as decayed. Modified by V. Anttonen from the image published in the Finnish Current Care Guidelines for Controlling Dental Caries (2014).

Using light of the dental unit (Planmeca Compact™ i Classic), an oral mirror, and a ball-point dental probe, caries lesions were examined visual-tactilely from 5 tooth surfaces of all except wisdom teeth, after drying them with a 3-in-1 syringe (World Health Organization 1997). FOTI was used routinely to transilluminate the tooth surfaces (DiaLUX probe 2300 L, KaVo). Before the examination, dental plaque was removed with a probe, if necessary.

For estimating the depth and activity (±) of caries lesions, the following ICDAS criteria (ICDAS Coordinating Committee 2012) were used: sound surface = 0, enamel/initial lesion = 1, 2 and inactive score 3- (I), lesions needing restorative treatment = active score 3+ and 4–6 (decayed, D) (Fig.). According to the clinical examination using the ICDAS criteria, DMFT, DMFS, and number of initial lesions were calculated.

#### Caries experience

At all time points, the number of initial and dentinal caries lesions on surfaces were summed up into main outcome variable, describing the total number of tooth surfaces affected by caries, called “caries experience” (I+DMFS/dmfs).

### Assessment of Dietary and Eating Habits

Eating frequency, energy and nutrient intake, and food consumption were assessed by food records. The records covered 4 predefined and consecutive days, including 2 weekdays and weekend days or 3 weekdays and 1 weekend day. Two food records at 2-y follow-up covering only 3 d (2 weekdays and 1 weekend day) were also included in the study (Lakka et al. 2020). Clinical nutritionists examined the filled food records together with the family at baseline and 2-y follow-up and with the adolescent at 8-y follow-up and added any missing information. Breakfast, lunch, and dinner were classified as main meals, and all eating and drinking occasions between them as snacks. The number of main meals and snacks per day were recorded (Eloranta et al. 2011). Food consumption and nutrient intake were assessed using the Micro Nutrica® dietary analysis software, version 2.5, based on detailed information about the nutrient content of foods in Finland and elsewhere (Eloranta et al. 2016). The intake of energy, energy nutrients, vitamins, and minerals were calculated from all their dietary sources. The use of vitamin and mineral supplements was not included in these analyses. Overall diet quality was assessed using the Baltic Sea Diet Score (BSDS) (Kanerva et al. 2014). BSDS was calculated by summing the scores of 6 components of food consumption and 2 components of nutrient intake in quartiles in the present population. The 6 components of food consumption were fruits and berries (scored 0–3), vegetables excluding potatoes (0–3), high-fiber (≥5%) grain products (0–3), low-fat (<1%) milk (0–3), fish (0–3), and red meat and sausages (3–0). Two components of nutrient intake were total fat intake as a percentage of total energy intake (3–0) and the ratio of polyunsaturated fatty acids to saturated fatty acids (0–3) (Kanerva et al. 2013). BSDS ranged between 0 and 24, and a higher score indicated better adherence to the diet.

At baseline and 2-y follow-up, eating behaviors were assessed using the Children’s Eating Behaviour Questionnaire (CEBQ) (Wardle et al. 2001) and at 8-y follow-up using the Adult’s Eating Behaviour Questionnaire (AEBQ) (Hunot et al. 2016). The CEBQ contains 35 questions and divides eating behaviors into 8 subscales: enjoyment of food, food responsiveness, satiety responsiveness, slowness in eating, emotional undereating, food fussiness, desire to drink, and emotional overeating. In AEBQ, the desire to drink is replaced with the hunger scale being otherwise the same as the CEBQ (Hunot et al. 2016). In both surveys, the subscales consisted of 3 to 6 statements, each rated on a 5-point Likert scale (*never* = 1, *rarely* = 2, *sometimes* = 3, *often* = 4, *always* = 5). The scores of the statements that belonged to the same subscale were summed, and the means and standard deviations were calculated. A higher mean value in the subscale indicated a greater likelihood for the given eating behavior trait.

### Assessment of Body Size, Toothbrushing Frequency, and Household Income

At all time points, body weight was measured twice after overnight fasting, bladder emptied, and in light underwear using a calibrated InBody^®^ 720 bioelectrical impedance device (Biospace). The means of these 2 values were used in the analyses. Body height was measured 3 times in the Frankfurt plane without shoes by a wall-mounted stadiometer. The mean of the 2 nearest values was used in the analyses. Body mass index (BMI) was calculated as body weight divided by body height (kg/m^2^). The BMI standard deviation score (BMI-SDS) was computed using the Finnish national references.

The frequency of toothbrushing (at least twice a day/once a day or less often) and household income (≤30 000€/30 001–60 000€/≥60 001€) were assessed by a questionnaire filled out by the parents or participants.

### Statistical Methods

The statistical analyses were performed using IBM SPSS Statistics software, version 27.0 (IBM Corp.). The associations of changes in eating frequency, energy and nutrient intake, diet quality, food consumption, and eating behavior with a change in caries experience over 8 y were examined using a generalized linear mixed-effects models (GLMM). The mixed-model structure was chosen as it can take account of the repeated measures–type structure in the data and uses all available information in the analysis and does not remove the whole study subject in the analysis if one measured point is missing. Separate GLMMs adjusted for the fixed effects of age, gender, study group (intervention vs. control), and household income at all time points and the repeated effect of time were performed to test the associations of each variable with caries experience (model 1). In model 2, GLMMs were additionally adjusted for BMI-SDS since the additional adjustment increased the model adequacy and affected the strength of the associations between dietary factors and a change in caries experience (Willerhausen et al. 2007). The association of change in calcium intake with a change in caries experience was further adjusted with consumption of sour milk. The random subject-specific intercept was used in the models. The equations for model 1 and 2 are given by

Model 1: OUTCOME_it_ = (*β*_0_ + u_i_ + *v_it_*) + *β*_1_age + *β*_2_gender+ *β*_3_study group + *β*_4_household income + *ε*_it_,
Model 2: OUTCOME_it_ = (*β*_0_ + u_i_ + *v_it_*) + *β*_1_age + *β*_2_gender + *β*_3_study group + *β*_4_household income + *β*_5_BMI-SDS + *ε*_it_, where OUTCOME_it_ are observations for subject *i* at baseline, 2-y follow-up, and 8-y follow-up; *β*_0_ is the intercept; *β*_1_, *β*_2_, *β*_3_, *β*_4_, and *β*_5_ are the regression coefficients for *age*, *gender*, *study group, household income*, and *BMI-SDS*, respectively; *u_i_* are random, subject-specific intercepts; *v_it_* is the random effect accounting for the repeated effects of time; and *ε_it_* is the error for subject *i* at time *t*. A Bayesian information criterion was used to measure model adequacy and find the best predicting parameters for the change of caries experience, with a lower value indicating a better model. We prioritized choosing a model with an optimal balance between good fit and complexity. We also tested models allowing dependent variable clustering on a subject within the school level. However, because the 3-level structure resulted in unnecessary complexity and did not improve model fit, clustering within schools was not considered in the final models.

### Ethical Considerations

Participation was voluntary, and no compensation was given to the participants. Data were collected and analyzed without personal identification information. The parents or caregivers of the children at baseline and the adolescents at 8-y follow-up provided their written consent, and the children at baseline gave their assent to participation. The study protocol was approved by the Research Ethics Committee of the Hospital District of Northern Savo in 2006 (statement 69/2006) and in 2015 (statement 422/2015).

## Results

The characteristics of participants at all 3 time points are presented in Table 1. Eating frequency, energy and nutrient intake, diet quality, food consumption, and eating behavior at baseline, 2-y follow-up, and 8-y follow-up are presented in Table 2.

**Table 1.** Basic Characteristics of the Participants at Baseline, 2-y Follow-up, and 8-y Follow-up.

	Baseline	2-y Follow-up	8-y Follow-up
	(*n* = 487)	(*n* = 406)	(*n* = 202)
Gender, *n* (%)			
Girls	234 (48)	199 (49)	96 (48)
Boys	253 (52)	207 (51)	106 (52)
Study group, *n* (%)			
Intervention	296 (61)	244 (60)	129 (64)
Control	191 (39)	162 (40)	73 (36)
Age, y	7.6 (0.4)	9.6 (0.5)	16.5 (0.5)
BMI-SDS	−0.17 (1.1)	−0.13 (1.1)	−0.1 (1.0)
DMFT/dmft	1.59 (2.46)	1.49 (2.16)	2.37 (2.85)
DMFS/dmfs	2.60 (4.34)	2.46 (3.89)	4.03 (5.86)
Number of tooth surfaces with initial caries lesions in primary and permanent teeth	1.28 (1.86)	1.36 (1.97)	16.6 (13.3)
Number of restored and extracted (due to caries) tooth surfaces as well as those with initial and dentinal caries lesions in primary and permanent teeth	3.89 (5.15)	3.82 (4.70)	20.7 (17.2)
Tooth brushing frequency, *n* (%)			
At least twice a day	281 (58)	246 (66)	137 (73)
Once a day or less often	183 (42)	129 (34)	50 (27)
Household income, *n* (%)			
≤30 000€	101 (21)	63 (16)	13 (7)
30 001€ to 60 000€	200 (42)	141 (36)	42 (23)
≥60 001€	178 (37)	186 (48)	127 (70)

The values are presented as means (standard deviations) for continuous variables and numbers (percentages) for categorical variables. There were complete data for all variables other than toothbrushing frequency and household income for all participants. Data on these 2 variables were available for the following numbers of participants at baseline, 2-y follow-up, and 8-y follow-up, respectively: toothbrushing frequency for 464, 375, and 187 participants; household income for 479, 390, and 180 participants.

BMI-SDS, body mass index standard deviation score; DMFT, decayed, missing, and filled teeth; dmft, decayed, missing, and filled teeth; DMFS, decayed, missing and filled surface; dmfs, decayed, missing, and filled surfaces. Written in lowercase letters, dmft/dmfs refers to the primary dentition.

**Table 2.** Eating Frequency, Energy and Nutrient Intake, Diet Quality, Food Consumption, and Eating Behavior at Baseline, 2-y Follow-up, and 8-y Follow-up.

	Baseline	2-year Follow-up	8-y Follow-up
Eating frequency			
Main meals (number/day)	2.8 (0.26)	2.7 (0.29)	2.5 (0.38)
Snacks (number/day)	2.7 (0.88)	2.5 (0.80)	2.2 (0.88)
Energy and nutrient intake^a^			
Energy intake (kcal/day)	1,642 (307)	1,694 (351)	1,845 (511)
Carbohydrate (E%)	51.8 (5.08)	50.3 (5.09)	47.2 (7.21)
Sucrose (E%)	12.7 (3.67)	11.4 (3.92)	10.4 (4.98)
Protein (E%)	16.8 (2.46)	16.9 (2.58)	17.6 (3.62)
Total fat (E%)	30.0 (5.00)	31.5 (5.22)	33.9 (6.57)
PUFA (E%)	4.92 (1.25)	5.76 (1.76)	6.25 (1.94)
MUFA (E%)	9.96 (1.85)	10.9 (2.16)	11.9 (3.19)
SFA (E%)	12.2 (2.77)	12.0 (2.66)	12.5 (1.93)
Vitamin D (µg/day)	5.87 (2.16)	8.32 (3.92)	10.0 (5.35)
Vitamin C (mg/day)	86.5 (43.7)	86.6 (46.3)	87.5 (54.0)
Vitamin E (mg/day)	6.78 (1.96)	7.89 (2.77)	9.35 (3.93)
Calcium (mg/day)	1,172 (348)	1,176 (372)	1,119 (533)
Folate (µg/day)	191 (49.1)	190 (53.1)	214 (79.0)
Zinc (mg/day)	10.0 (2.09)	10.4 (2.49)	11.3 (3.83)
Iron (mg/day)	8.28 (2.04)	8.43 (2.18)	9.70 (3.55)
Fiber (g/day)	14.5 (4.06)	15.2 (4.62)	17.1 (6.58)
Diet quality			
Baltic Sea Diet Score (range 0–24)	11.8 (4.3)	12.0 (4.1)	12.1 (4.0)
Food consumption (g/d)			
High-fiber (≥5%) grain products^b^	63.3 (39.4)	75.1 (45.9)	85.5 (60.3)
Low-fiber (<5%) grain products^b^	113 (52.5)	108 (67.1)	118 (71.6)
Vegetables and nuts^c^	103 (58.5)	106 (65.3)	127 (72.9)
Potato	76.2 (41.8)	76.7 (53.1)	86.7 (65.0)
Fruits and berries	109 (83.9)	103 (82.1)	106 (110)
Butter and butter-oil mixtures	5.8 (7.1)	6.0 (8.7)	8.34 (9.46)
Vegetable oil-based margarine (60%–80% fat)	7.3 (8.1)	14.9 (14.8)	11.0 (12.9)
Vegetable oil–based margarine (<60% fat)	3.9 (6.9)	2.2 (6.3)	0.90 (4.8)
Vegetable oil	4.1 (4.2)	4.5 (4.9)	5.79 (5.49)
Milk (<1% and ≥1% fat)	567 (252)	563 (267)	447 (319)
Sour milk (<1% and ≥1% fat)^d^	104 (94.3)	85.5 (92.6)	73.3 (93.6)
Cheese	15.9 (15.9)	17.2 (15.8)	27.6 (25.5)
Fish and fish products^e^	16.0 (21.4)	18.2 (24.4)	20.5 (25.1)
Poultry	16.9 (21.6)	21.6 (25.3)	39.5 (39.7)
Red meat and sausages^f^	79.1 (38.9)	82.4 (45.1)	91.4 (64.2)
Sugar-sweetened beverages^g^	136 (128)	147 (137)	150 (184)
Artificially sweetened beverages^g^	42.8 (81.2)	59.8 (108)	80.2 (141)
Fruit juices	36.0 (67.4)	37.8 (72.2)	42.8 (73.2)
Pudding and ice cream	27.1 (31.4)	23.5 (28.5)	11.7 (20.8)
Sweets	20.9 (24.1)	23.7 (27.1)	19.2 (28.4)
Chocolate	9.7 (12.4)	11.0 (14.2)	11.8 (17.3)
Salty snacks^h^	4.2 (10.4)	6.7 (16.1)	7.5 (15.9)
Eating behavior			
Enjoyment of food (range 1–5)	3.2 (0.7)	3.2 (0.7)	4.2 (0.7)
Food responsiveness (range 1–5)	1.7 (0.6)	1.7 (0.6)	2.6 (0.8)
Satiety responsiveness (range 1–5)	3.0 (0.6)	2.9 (0.6)	2.5 (0.8)
Slowness in eating (range 1–5)	2.7 (0.8)	2.5 (0.7)	2.6 (0.8)
Emotional undereating (range 1–5)	2.5 (0.9)	2.8 (0.6)	2.2 (0.9)
Food fussiness (range 1–5)	2.8 (0.8)	2.8 (0.8)	2.2 (0.7)
Emotional overeating (range 1–5)	1.4 (0.5)	1.5 (0.5)	2.1 (0.9)

The values are presented as means (standard deviations). Data on other variables than those related to eating behaviors were available for the following numbers of participants at baseline, 2-y follow-up, and 8-y follow-up, respectively: eating frequency, energy and nutrient intake, and food consumption for 413, 359, and 182 participants; Baltic Sea Diet Score for 400, 331, and 184 participants. Data on eating behaviors were available for 484 to 473 participants at baseline, for 395 to 391 participants at 2-y follow-up, and for 189 to 187 participants at 8-y follow-up.

MUFA, monounsaturated fatty acid; PUFA, polyunsaturated fatty acid; SFA, saturated fatty acid.

a E% indicates the percentage of total energy intake.

b Includes bread, cereal, porridge, flour, pasta, and rice.

c Includes vegetables, nuts, roots, beans, and mushrooms, excluding potato.

d Includes yoghurt, curdled milk, sour milk, and quark.

e Includes fish, shellfish, and fish products.

f Includes pork, beef, lamb, reindeer, game meat, and sausages.

g Includes carbonated and noncarbonated beverages.

h Includes popcorn, crisps, and salted nuts.

During the 8-y follow-up, an increased number of snacks, intake of calcium, and consumption of sour milk products and salty snacks per day were associated with increased caries experience when adjusted for age, gender, household income, and study group (Table 3, model 1). Further adjustment for BMI-SDS had no effect on the associations of the number of snacks, the intake of calcium, and the consumption of salty snacks with caries experience. The association between consumption of sour milk products and increased caries experience had almost negligible alteration with further adjustment for BMI-SDS (Table 3, model 2). The association between calcium intake and caries experience attenuated after further adjustment for sour milk products (Table 3, models 1 and 2).

During the 8-y follow-up, increased BSDS and consumption of butter and butter-oil mixtures were associated with decreased caries experience (Table 3, model 1). The association between BSDS and caries experience weakened and was no longer statistically significant after further adjustment for BMI-SDS, whereas further adjustment for BMI-SDS had no effect on the association between butter and butter-oil mixtures and caries experience (Table 3, model 2).

**Table 3.** Associations of Changes in Dietary Factors with an Increase in Caries Experience over 8 y.

	Model 1*		Model 2**	
	Fixed Coefficient	*P* Value	Fixed Coefficient	*P* Value
	(95% CI)		(95% CI)	
Eating frequency				
Main meals (number/d)	0.060 (−0.150, 0.270)	0.574	0.067 (−0.142, 0.276)	0.530
Snacks (number/d)	0.072 (0.006, 0.139)	**0.032**	0.077 (0.011, 0.142)	**0.023**
Energy and nutrient intake^a^				
Energy intake (kcal/d)	9.63 × 10^−5^ (−8.69 × 10^−5^, 2.79 × 10^−4^)	0.302	8.46 × 10^−4^ (−9.83 × 10^−5^, 2.68 × 10^−4^)	0.364
Carbohydrate (E%)	−0.008 (−0.021, 0.005)	0.237	−0.007 (−0.019, 0.006)	0.317
Sucrose (E%)	0.012 (−0.005, 0.030)	0.175	0.013 (−0.005, 0.031)	0.148
Protein (E%)	0.007 (−0.018, 0.033)	0.582	0.006 (−0.019, 0.032)	0.624
Total fat (E%)	0.006 (−0.007, 0.018)	0.380	0.005 (−0.008, 0.017)	0.462
PUFA (E%)	0.013 (−0.028, 0.055)	0.526	0.013 (−0.028, 0.054)	0.544
MUFA (E%)	0.004 (−0.027, 0.035)	0.791	0.004 (−0.027, 0.034)	0.823
SFA (E%)	0.014 (−0.008, 0.037)	0.214	0.010 (−0.005, 0.026)	0.184
Vitamin D (µg/d)	−0.003 (−0.022, 0.016)	0.765	−0.003 (−0.021, 0.016)	0.784
Vitamin C (mg/d)	−0.001 (−0.002, 4.49 × 10^−4^)	0.195	−0.001 (−0.002, 3.35 × 10^−4^)	0.144
Vitamin E (mg/d)	−0.001 (−0.028, 0.027)	0.971	−0.001 (−0.028, 0.027)	0.959
Calcium (mg/d)	2.41 × 10^−4^ (3.46 × 10^−5^, 4.47 × 10^−4^) 1.88 × 10^−4^ (−2.946 × 10^−5^, 4.06 × 10^−4^)***	**0.022** 0.090***	2.22 × 10^−4^ (1.58 × 10^−4^, 4.29 × 10^−4^) 1.74 × 10^−4^ (−4.438 × 10^−5^, 3.92 × 10^−4^)***	**0.035** 0.118***
Folate (µg/d)	−0.001 (−0.002, 0.001)	0.303	−0.001 (−0.002, 0.001)	0.269
Zinc (mg/d)	−0.006 (−0.037, 0.026)	0.725	−0.006 (−0.037, 0.026)	0.725
Iron (mg/d)	0.010 (−0.021, 0.042)	0.521	0.010 (−0.021, 0.042)	0.528
Fiber (g/d)	−0.007 (−0.023, 0.008)	0.357	−0.008 (−0.024, 0.007)	0.297
Diet quality				
Baltic Sea Diet Score	−0.017 (−0.035, −3.46 × 10^−4^)	**0.046**	−0.016 (−0.033, 0.002)	0.076
Food consumption (g/d)				
High-fiber (≥5%) grain products^b^	−0.001 (−0.002, 0.001)	0.332	−0.001 (−0.002, 0.001)	0.315
Low-fiber (<5%) grain products^b^	3.97 × 10^−4^ (−0.001, 0.001)	0.450	−3.69 × 10^−4^ (−0.001, 0.001)	0.482
Vegetables and nuts^c^	−4.64 × 10^−4^ (−0.002, 0.001)	0.410	−0.001 (−0.002, 0.001)	0.372
Potato	0.001 (−0.001, 0.002)	0.400	4.09 × 10^−4^ (−0.001, 0.002)	0.531
Fruits and berries	−6.36 × 10^−5^ (−0.001, 0.001)	0.870	−1.07 × 10^−4^ (−0.001, 0.001)	0.783
Butter and butter-oil mixtures	−0.009 (−0.017, −2.26 × 10^−4^)	**0.044**	−0.009 (−0.017, −4.90 × 10^−4^)	**0.038**
Vegetable oil-based margarine (60%–80% fat)	0.001 (−0.005, 0.006)	0.747	0.001 (−0.004, 0.007)	0.642
Vegetable oil–based margarine (<60% fat)	−0.006 (−0.016, 0.005)	0.292	−0.008 (−0.018, 0.003)	0.140
Vegetable oil	0.001 (−0.014, 0.017)	0.878	0.002 (−0.013, 0.018)	0.786
Milk (<1% and ≥1% fat)	2.12 × 10^−4^ (−7.38 × 10^−5^, 4.97 × 10^−4^)	0.146	2.15 × 10^−4^ (−6.96 × 10^−5^, 4.99 × 10^−4^)	0.139
Sour milk products (<1% and ≥1% fat)^d^	0.001 (3.81 × 10^−5^, 0.001)	**0.039**	0.001 (1.09 × 10^−5^, 0.001)	0.054
Cheese	−0.001 (−0.005, 0.004)	0.737	−0.002 (−0.006, 0.003)	0.462
Fish and fish products^e^	−0.001 (−0.003, 0.001)	0.448	−0.001 (−0.003, 0.002)	0.600
Poultry	−0.001 (−0.003, 0.002)	0.550	−0.001 (−0.003, 0.002)	0.543
Red meat and sausages^f^	0.001 (−0.001. 0.002)	0.333	0.001 (−0.001, 0.002)	0.418
Sugar-sweetened beverages^g^	−1.05 × 10^−4^ (−0.001, 4.22 × 10^−4^)	0.696	−9.06 × 10^−5^ (−0.001, 4.35 × 10^−4^)	0.735
Artificially sweetened beverages^g^	2.94 × 10^−4^ (−3.73 × 10^−4^, 0.001)	0.387	3.30 × 10^−4^ (−3.34 × 10^−4^, 0.001)	0.329
Fruit juices	1.92 × 10^−4^ (−0.001, 0.001)	0.663	1.87 × 10^−4^ (−0.001, 0.001)	0.669
Pudding and ice cream	0.001 (−0.001, 0.003)	0.228	0.001 (−0.001, 0.003)	0.175
Sweets	−3.13 × 10^−4^ (−0.003, 0.002)	0.823	−3.63 × 10^−5^ (−0.003, 0.003)	0.979
Chocolate	0.002 (−0.002, 0.006)	0.366	0.002 (−0.002, 0.006)	0.395
Salty snacks^h^	0.006 (0.001, 0.011)	**0.010**	0.006 (0.001, 0.011)	**0.016**
Eating behavior				
Enjoyment of food	−0.121 (−0.240, −0.002)	**0.046**	−0.122 (−0.241, −0.003)	**0.044**
Food responsiveness	0.025 (−0.099, 0.149)	0.692	−0.012 (−0.140, 0.116)	0.853
Satiety responsiveness	0.060 (−0.062, 0.182)	0.332	0.057 (−0.065, 0.178)	0.361
Slowness in eating	0.113 (−0.001, 0.226)	**0.051**	0.119 (0.006, 0.232)	**0.038**
Emotional undereating	−0.068 (−0.176, 0.040)	0.217	−0.067 (−0.174, 0.040)	0.219
Food fussiness	0.140 (0.035, 0.245)	**0.009**	0.135 (0.030, 0.240)	**0.012**
Emotional overeating	0.076 (−0.052, 0.204)	0.244	0.065 (−0.063, 0.193)	0.322

Caries experience includes DMFS, dmfs, and initial caries surfaces in primary and permanent teeth. The values are fixed coefficients, their 95% confidence intervals, and *P* values from generalized linear mixed-effects models, adjusted for the fixed effects of gender, study group (intervention vs. control), and household income at both time points and the repeated effect of time (model 1*). In model 2**, BMI-SDS is also adjusted. Intake of calcium was further adjusted for sour milk products in both models 1 and 2***. The random subject-specific intercept was used in the models. *P* values <0.05 are bolded. Data on variables were available for the following numbers of children at baseline, 2-y follow-up, and 8-y follow-up, respectively: caries experience 487, 406, and 202; eating frequency, energy and nutrient intake, and food consumption variables 413, 359, and 182. Eating behavior variables varied from 484 to 473 at baseline, 395 to 391 at 2-y follow-up, and 189 to 187 at 8-y follow-up.

BMI-SDS, body mass index standard deviation score; CI, confidence interval; MUFA, monounsaturated fatty acid; PUFA, polyunsaturated fatty acid; SFA, saturated fatty acid.

a E% indicates the percentage of total energy intake.

b Includes bread, cereal, porridge, flour, pasta, and rice.

c Includes vegetables, nuts, roots, beans, and mushrooms, excluding potato.

d Includes yoghurt, curdled milk, sour milk, and quark.

e Includes fish, shellfish, and fish products.

f Includes pork, beef, lamb, reindeer, game meat, and sausages.

g Includes carbonated and noncarbonated beverages.

h Includes popcorn, crisps, and salted nuts.

During the 8-y follow-up, increased enjoyment of food was associated with decreased caries experience, whereas increased slowness in eating and food fussiness were associated with increased caries experience (Table 3, model 1). These associations remained similar after further adjustment for BMI-SDS (Table 3, model 2).

## Discussion

This study showed an association of improved diet quality and enjoyment of food with decreased caries experience over 8 y from childhood to adolescence, which is novel information. Increased number of snacks and consumption of sour milk products and salty snacks were associated with increased caries experience. In addition, increased calcium intake was associated with increased caries experience, but this association was explained by the consumption of sour milk products. On the other hand, increased consumption of butter and butter-oil mixtures was associated with decreased caries experience. Increased enjoyment of food was associated with decreased caries experience, whereas increased slowness in eating and food fussiness were associated with increased caries experience.

The observed association of increased number of snacks, especially salty snacks, with increased caries experience from childhood to adolescence is in line with the results of other studies among children followed up for a period of 2 to 5 y (Hancock et al. 2020). According to the Finnish national nutrition and food recommendations for school-aged children, smartly selected snacks and regular mealtimes, including breakfast, lunch, and dinner, are recommended (Finnish Food Authority 2019). The average eating frequency of school-aged children in our study was consistent with the recommendations. However, the number of main meals and, interestingly, also snacks was slightly smaller in adolescence. It is possible that the number of snacks at 8-y follow-up was underreported, as shown previously, especially among those with frequent snacking (Tooze et al. 2004). Salty snacks are often highly processed, high in saturated fatty acids, and low in nutrients and are often consumed with beverages or other sweet products (Johansson et al. 2010). Potato chips contain highly processed starch, sticking easily to teeth. A combination of highly processed starch and sucrose has been shown to increase sucrose cariogenicity and even surpass it (Giacaman 2018). Earlier, Eloranta et al. (2011) reported that snacks comprised even two-thirds of the daily sucrose intake at the age of 6 to 8 y in this study population. These facts can explain the association between snacking and caries experience in our study. However, BMI-SDS did not explain the association. Snacking has also been associated with other detrimental oral health habits, including smoking and infrequent toothbrushing in young adults (Tanner et al. 2020).

An interesting finding of our study was the association of increased consumption of butter and butter-oil mixtures with decreased caries experience from childhood to adolescence, which is in line with the results of some previous studies demonstrating an anticariogenic effect of fats (Giacaman 2018). However, the underlying mechanism for the association remains unclear. Certain fatty acids have been shown to reduce the effect of sugars on the caries process (Giacaman 2018). Introducing certain unsaturated fatty acids, oleic and linoleic acids, to *Streptococcus mutans* biofilms following a sucrose exposure disrupted *S. mutans* biofilm accumulation and decreased enamel demineralization. However, stearic acid, a saturated fatty acid, showed no impact on the biofilm (Giacaman et al. 2015). In butter-oil mixtures, the content of unsaturated fatty acids is higher than in butter and can somewhat explain our result. Moreover, it has been previously reported that main meals contain more fat than snacks among children (Eloranta et al. 2011), which may also partly explain our finding. It is likely that butter and butter-oil mixtures were mainly used as spread on bread. Thus, the consumption of butter could indicate the consumption of high-fiber grain products, which have previously been associated with lower caries experience (Virkkala et al. 2022). However, we found no association between the change in high-fiber grain products and the change in caries experience. Further research in humans is warranted to explore this topic, for example, the content of these products, the time of day they are consumed, and any other products consumed alongside them.

Surprisingly, increased consumption of sour milk products and calcium intake were associated with increased caries experience from childhood to adolescence. The unexpected result concerning calcium intake was suggested to indicate consumption of sweet dairy products. Therefore, this association was further adjusted for consumption of sour milk products. The association attenuated and is thus explained by the consumption of sour milk products. Milk products contain cariostatic factors such as calcium, phosphate, casein, other protein components (Moynihan 2007), and fat (Giacaman 2018) and are therefore considered healthy for the teeth. However, sucrose is added to many sour milk products, such as yoghurts, which can explain their cariogenicity (Bowen and Pearson 1993; Ricomini Filho et al. 2021). It has been previously reported that sour milk products are one of the major sources of sucrose among Finnish children, providing 14.5% of sucrose intake (Eloranta et al. 2016). Particularly, if sugar-sweetened dairy products are consumed on their own between meals, they can have a more harmful effect on teeth than when consumed as part of a meal. The role of lactose in milk’s cariogenicity has been debated, but it has been shown to be low (Bowen and Pearson 1993). In addition, milk’s other constituents, such as minerals and casein, might overcome the harmful effects of lactose (Bowen and Pearson 1993).

The association of improved diet quality with decreased caries experience was partly explained by BMI-SDS. A similar association was reported earlier at 2-y follow-up, where the association was also attenuated after controlling for adiposity (Virkkala et al. 2022). The BSD is rich in foods grown and produced in Nordic countries, such as berries, vegetables, whole grains, low-fat milk products, rapeseed oil, and fish and low in processed meat products and alcohol (Kanerva et al. 2014). The recommended diet covers the need for nutrients, and only vitamin D supplementation is recommended year-round for all children and adolescents younger than 18 y in Finland (Finnish Food Authority 2019). Adherence to the healthy Nordic diet has been shown to be associated, for example, with a decreased risk of obesity (Kanerva et al. 2013). Our findings suggest that healthy diets such as BSD play an important role not only in overall health but also in oral health.

Associations between CEBQ subscales and caries experience have been studied in only a limited number of cross-sectional studies with varying results (Anandakrishna et al. 2014; Nembhwani and Winnier 2019; Shqair et al. 2022), which adds to the value of this study. Enjoyment of food has been associated with healthy dietary habits, such as regular meals (Jalkanen et al. 2017), which can partly explain the findings here. Among children, fussy eating has been related to a lower consumption of vegetables and protein-rich foods, such as cheese and meat (Jalkanen et al. 2017). Anandakrishna and coworkers (2014) found that problematic eating behaviors related to an increased caries risk can be seen already at the age of 3 y, which is somewhat in line with the findings here. In adults, fussy eating has been associated with a lower consumption of fruits and vegetables and a higher consumption of desserts, snacks, and beverages (Zickgraf and Schepps 2016).

In caries diagnostics, FOTI was used as an adjunct method to visual inspection. We did not take BW radiographs, and for screening purposes, BW are not recommended according to Finnish guidelines (Duodecim 2023). FOTI has lower sensitivity than specificity, making false-positive findings rare (Twetman et al. 2013). In epidemiological studies, FOTI’s reproducibility has been shown to be good (da Silva et al. 2011). Although research-based evidence on the benefits of using FOTI is limited, it can support observations made during clinical visual examinations (Duodecim 2023).

The strengths of our study include a relatively large, population-based sample of children followed up for 8 y until adolescence. We also had comprehensive data on dietary factors and eating behaviors. Experienced dentists performed oral health examinations at baseline, at 2-y follow-up, and at 8-y follow-up. ICDAS was used at the 8-y follow-up. At baseline and the 2-y follow-up, the World Health Organization classification using the DMFT/dmft and DMFS/dmfs indices was used. Clinical examinations by general practitioners and trained examiners have been found comparable, and consequently, data from patient records have been found reliable to be used for scientific purposes (Hausen et al. 2001). This justifies the use of data on examinations at baseline and at 2-y follow-up by experienced dentists. Moreover, we had data on oral self-care habits reported by the adolescents at 8-y follow-up or by their parents at baseline and at 2-y follow-up. Data on household income were available at all 3 time points as well. We also assessed eating behaviors using internationally validated questionnaires.

With regard to study limitations, the children participated in a physical activity and dietary intervention study. Therefore, families involved in the study may have been more health-oriented than the general population. The children were allocated to the intervention and the control group, which might also have influenced our results but was also controlled in the analyses. Nutrient intake and food consumption were measured using food records, which can be prone to inaccuracies (Shim et al. 2014). A clinical nutritionist grouped the food items, which might have influenced the rankings. When detailed information was not available, standard recipes were used for homemade foods. This could have caused nutrient intake to be overestimated or underestimated for individuals. However, this most likely had a minor effect at the group level (Eloranta et al. 2016). The high standard deviation observed in the consumption of certain food items, particularly sour milk products, indicates considerable variation in dietary habits within this limited study cohort, thus decreasing the generalization of the outcome. Finally, during the 8-y follow-up period, 285 participants were lost from the study, compared with 227 in the nondental modules. In long-term studies, participant relocation is common, but other reasons for dropout include low motivation and dental anxiety. Furthermore, at baseline and the 2-y follow-up, PANIC dental examinations replaced the children’s regular dental checkups, which were tailored to their age and individual needs. However, at the 8-y follow-up, these examinations no longer replaced regular dental visits, requiring participants to be highly motivated to attend repeated dental assessments. Another potential reason for dropout is that PANIC dental examinations were conducted separately, after extensive and time-consuming nondental modules, which could contribute to loss through physical and mental fatigue. Missing data may have limited statistical power in some analyses. However, attrition was accounted for in the power calculations conducted at the 2-y follow-up (Lakka et al. 2020). Also, a mixed model was used, which can account for the fact that not all participants have results at every time point.

## Conclusion

We demonstrated here how vital a healthy diet is for oral health among growing children when controlling for adiposity. We also showed that eating behaviors and enjoyment of food play a role in maintaining good oral and eating health. Since diet behaviors developing from childhood to adolescence were associated with changes in caries experience, providing individualized dietary counseling to children and their caregivers would be essential.

## Author Contributions

M. Methuen, V. Anttonen, T.A. Lakka, A.L. Suominen, A.-M. Eloranta, contributed to conception, design, data acquisition and interpretation, drafted and critically revised the manuscript; V.F. Kukkonen, contributed to conception, design, data analysis and interpretation, drafted and critically revised the manuscript; S. Mikkonen, contributed to data analysis, critically revised the manuscript; J. Väistö, contributed to data acquisition and analysis, critically revised the manuscript; S. Soininen, M. Närhi, contributed to data acquisition, critically revised the manuscript. All authors gave final approval and agree to be accountable for all aspects of the work.
